# Supplementary material for: Label-free pathological subtyping of non-small cell lung cancer using deep classification and virtual immunohistochemical staining
Source: NPJ Digit Med. 2026 Apr 3;9:438. doi: 10.1038/s41746-026-02557-x (PMC13249941; doi:10.1038/s41746-026-02557-x)
Supplement: Supplementary file 1 — Supplementary information [file 41746_2026_2557_MOESM1_ESM.pdf]

Supplementary information of "Label-free non-small cell lung cancer subtyping using deep classification and virtual IHC staining"

Zhenya Zang[1], David A Dorward[2], Katherine E Quiohilag[2], Andrew DJ Wood[2], James R Hopgood[3], Ahsan R Akram[1†*], Qiang Wang[1†*]

[1]Centre for Inflammation Research, Institute of Regeneration and Repair, The University of Edinburgh, Edinburgh, UK.

[2]Department of Pathology, Royal Infirmary of Edinburgh, Edinburgh, UK.

[3]Insititue of Imaging, Data and Communications, School of Engineering, The University of Edinburgh, Edinburgh, UK.

† These authors jointly supervised the work and are co-senior authors

* Corresponding authors: Q.Wang@ed.ac.uk, Ahsan.Akram@ed.ac.uk

Supplementary figures

Figure 1. Binary classification for four cases using different DNN models.

Figure 2. An example core of the AC subtype with intensity, FLIM, and H&E-stained images.

Figure 3. An example core of the SqCC subtype with intensity, FLIM, and H&E-stained images.

Figure 4. An example core of the OS subtype with intensity, FLIM, and H&E-stained images.

Figure 5. A TMA core where the intensity-based virtual TTF-1 image is ambiguous for pathologists to make confident decisions.

Figure 6. A TMA core where both virtual p40 images are ambiguous for pathologists to make confident decisions.

Figure 7. Virtual IHC staining of a solid pattern NSCLC and surrounding lung parenchyma

Figure 8. Distributions of means and standard deviations for individual cores of four subtypes, within the test datasets.

Figure 9. H&E-stained images of AC's five subtypes, solid, lepidic, acinar, papillary, and micropapillary, involved in the datasets.

Figure 10. Subtyping and virtual staining results of biopsy 101.

Figure 11. Subtyping and virtual staining results of biopsy 106.

Figure 12. Overview of deep learning architectures for lung cancer subtyping and TTF-1 and p40 virtual staining.

Supplementary tables

Table 1. Clinical details on a patient level for the tissue microarrays used in this study.

Table 2. Number of patches and cores of each subtype in training, validation, and test datasets.

Table 3. Performance evaluation of classical deep learning architectures for multiple cancer type classification, using different metrics.

Table 4. Morphological AC subtypes used in this study.

Table 5. Ground-truth clinicopathological annotations of lung biopsy specimens, including histological diagnosis and differentiation pattern.

Table 6. Extensive Performance Evaluation of DenseNet for binary classification.

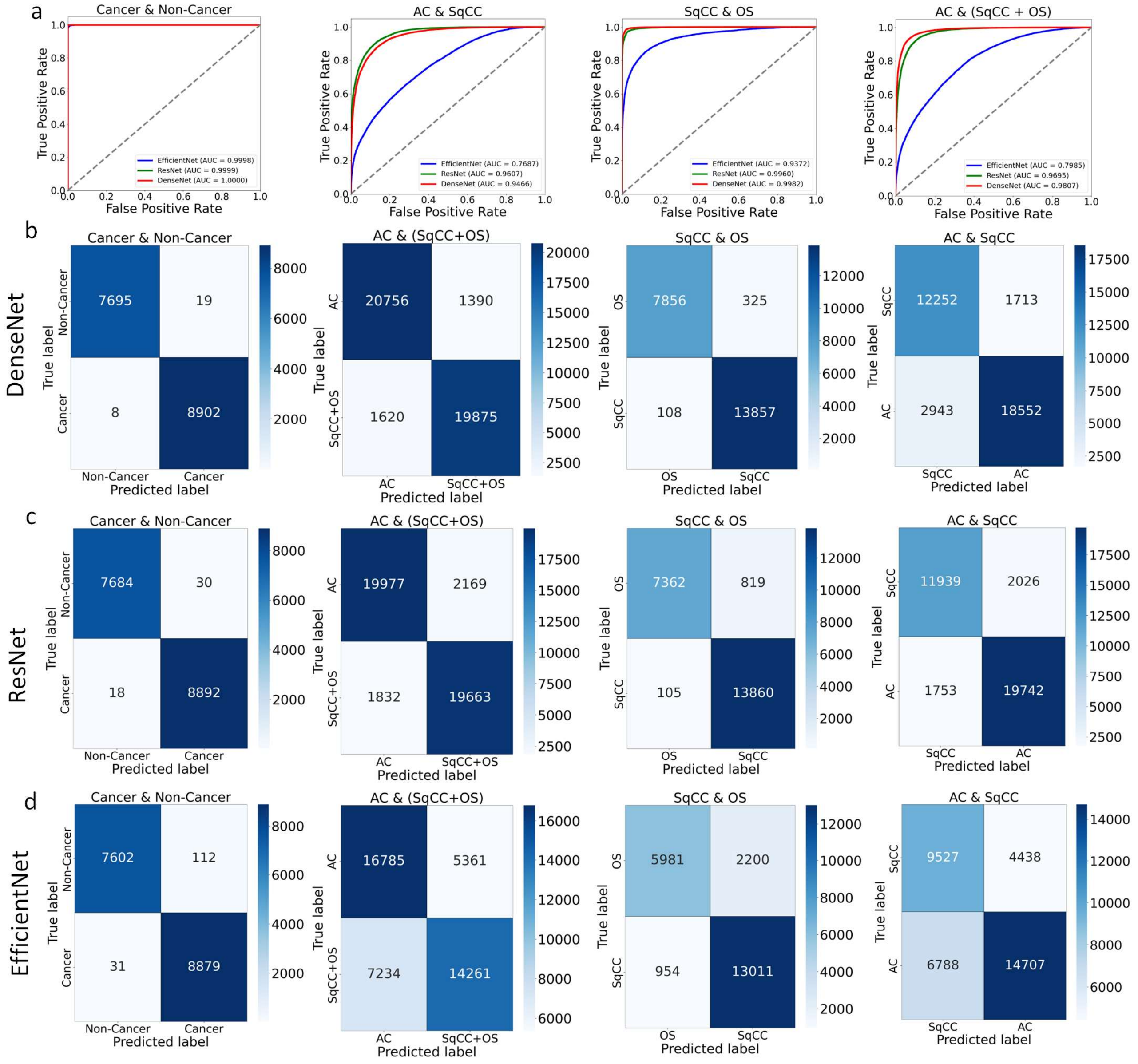

Supplementary Fig. S1. **Binary classification for four cases using different DNN models.** (a) ROCs and AUC scores, and (b), (c), and (d) confusion matrices from DenseNet, ResNet, and EfficientNet for four-class classification.

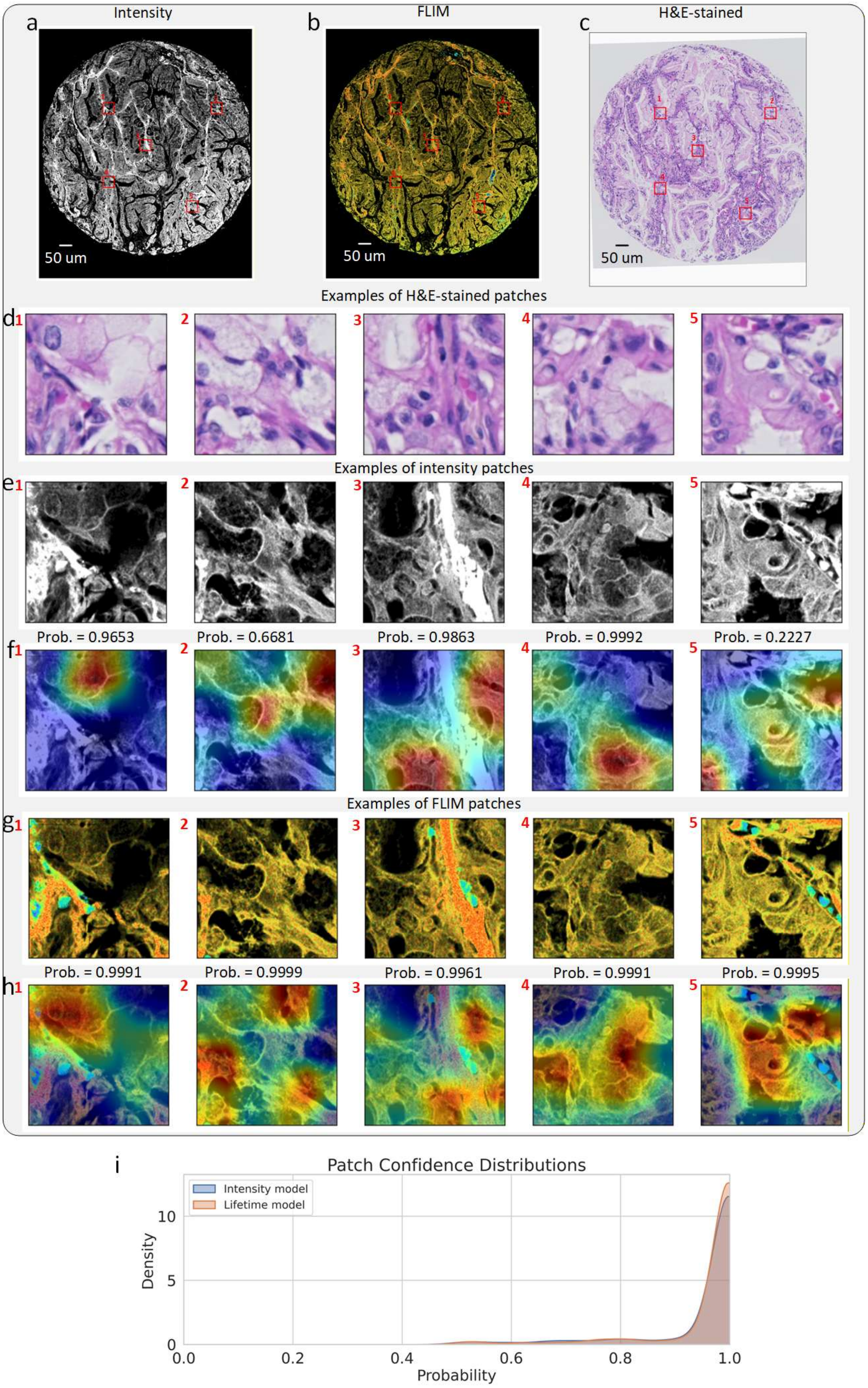

Supplementary Fig. S2. An example core of the AC subtype with intensity, FLIM, and H&E-stained images. (a-c) Five patches are highlighted as examples, showcasing different tissue components and morphologies. (d). H&E-stained images. (e) and (g) Inferred AC probabilities from the intensity- and FLIM-based DL model. (f) and (h) Salience maps generated by Grad-Cam++ from intensity- and FLIM-based models. (i) The probability distribution of intensity and lifetime models from patches in the core is presented.

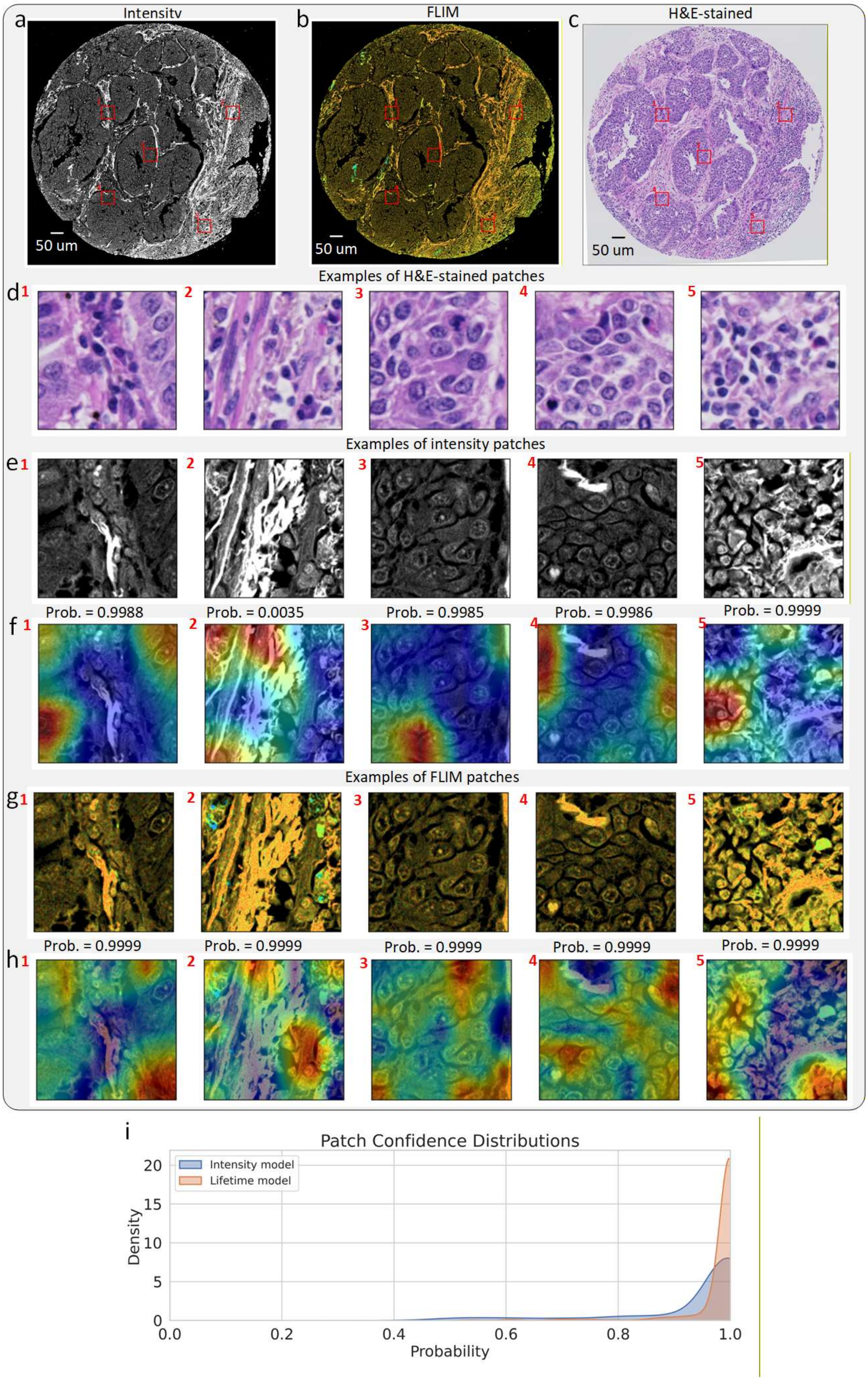

Supplementary Fig. S3. An example core of the SqCC subtype with intensity, FLIM, and H&E-stained images. (a-c) Five patches are highlighted as examples, showcasing different tissue components and morphologies. (d). H&E-stained images. (e) and (g) Inferred SqCC probabilities from the intensity- and FLIM-based DL model. (f) and (h) Salience maps generated by Grad-Cam++ from intensity- and FLIM-based models. (i) The probability distribution of intensity and lifetime models from patches in the core is presented.

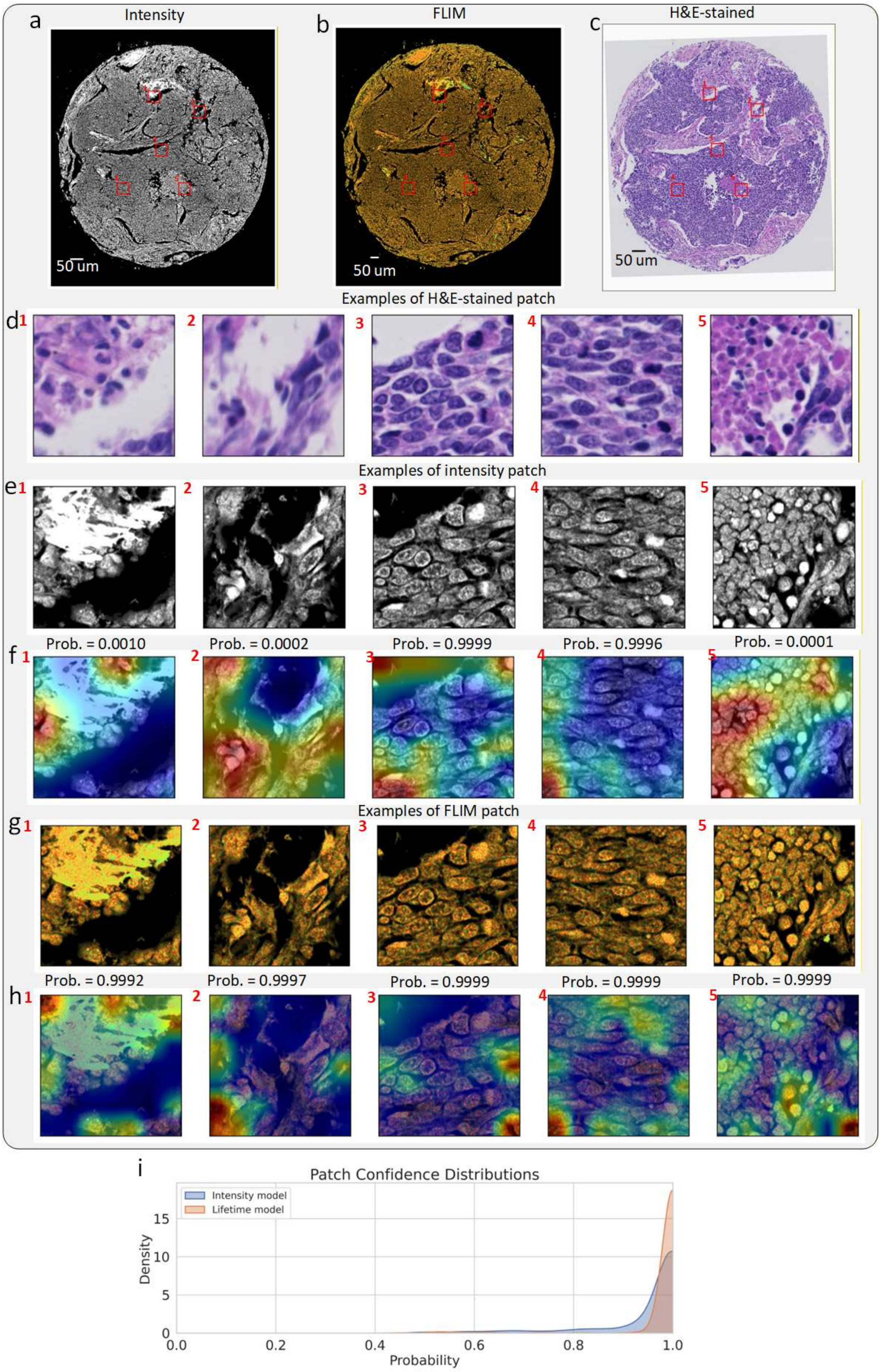

Supplementary Fig. S4. An example core of the OS subtype with intensity, FLIM, and H&E-stained images. (a-c) Five patches are highlighted as examples, showcasing different tissue components and morphologies. (d). H&E-stained images. (e) and (g) Inferred OS probabilities from the intensity- and FLIM-based DL model. (f) and (h) Salience maps generated by Grad-Cam++ from intensity- and FLIM-based models. (i) The probability distribution of intensity and lifetime models from patches in the core is presented.

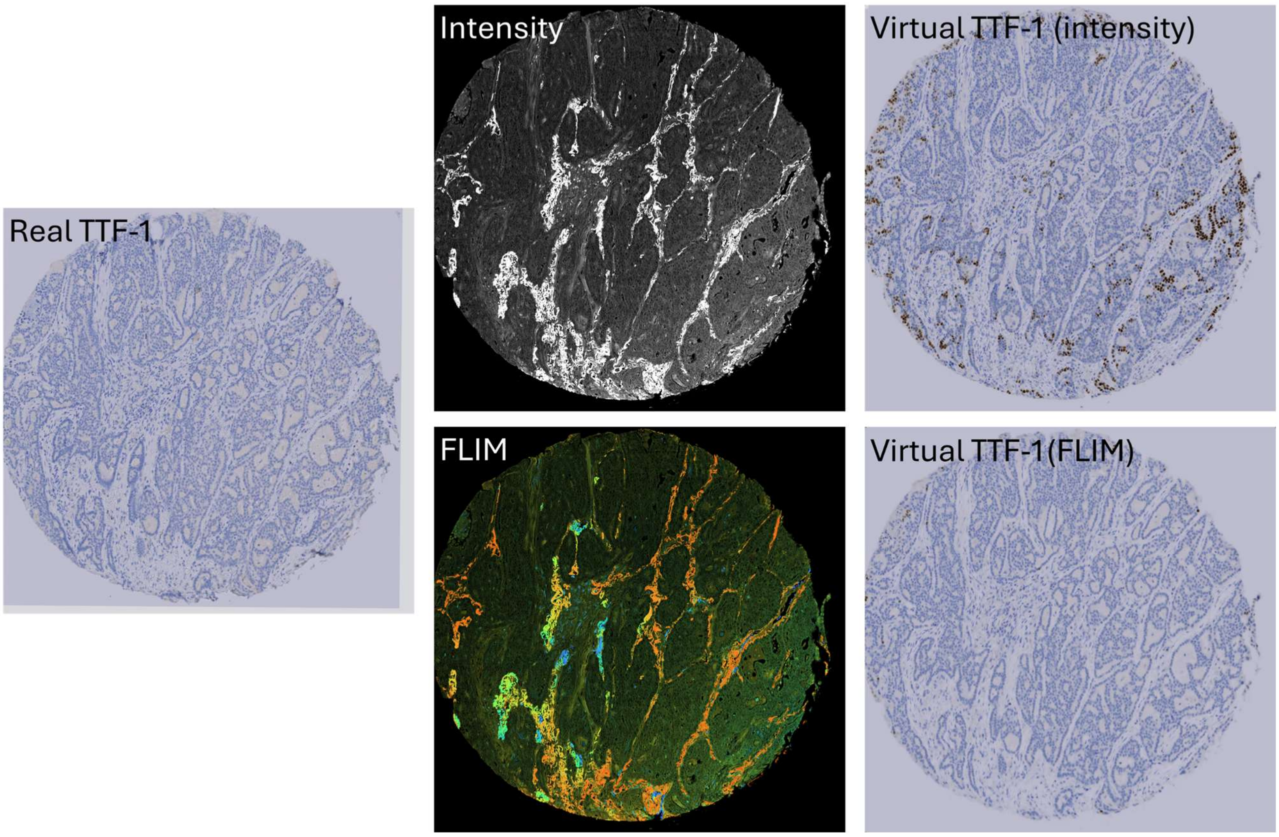

Supplementary Fig. S5. A TMA core where the intensity-based virtual TTF-1 image is ambiguous for pathologists to make confident decisions.

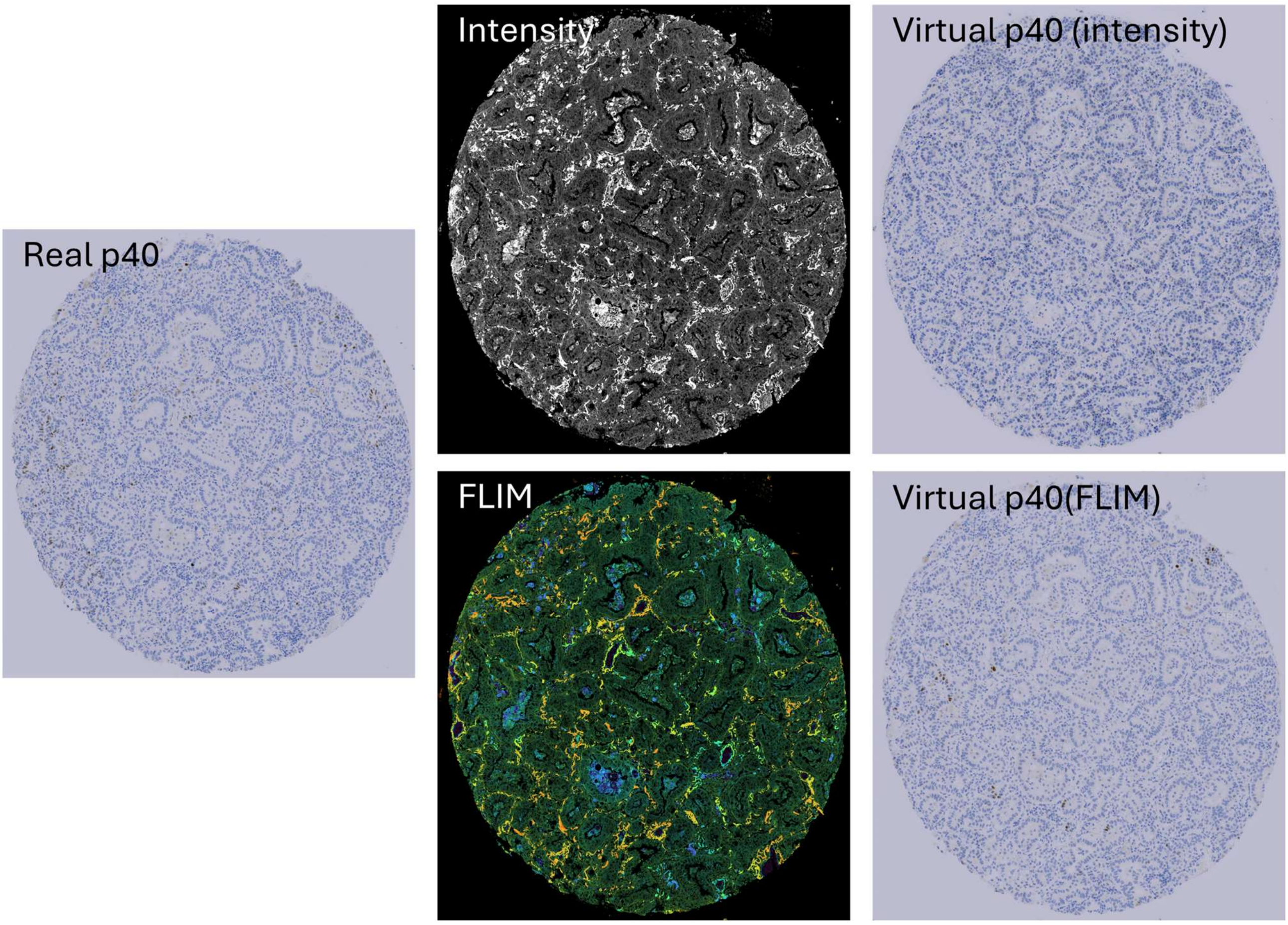

Supplementary Fig. S6: A TMA core where both virtual p40 images are ambiguous for pathologists to make confident decisions.

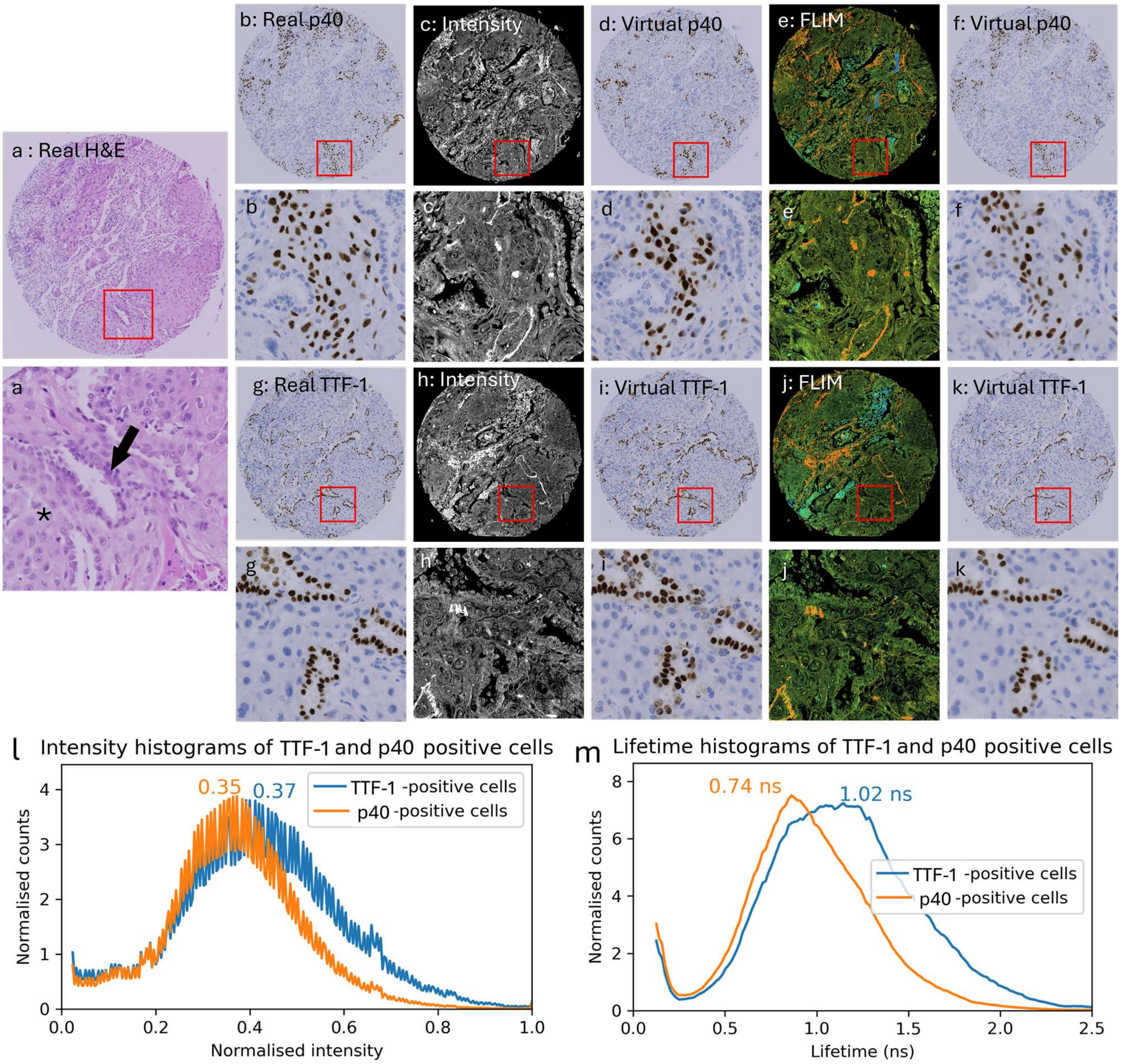

Supplementary Fig. S7: Virtual IHC staining of a solid pattern NSCLC and surrounding lung parenchyma. H&E stain (a) demonstrates solid nests of poorly differentiated malignant epithelial cells (*) with a squamoid morphology, which surround groups of entrapped, reactive alveolar pneumocytes (arrow). In a clinical context, p40 and TTF-1 IHC use is advisable for definitive subtyping. Here, both p40 (b) and TTF-1 (g) are expressed, with p40 expression within the malignant cells confirming the morphological impression of squamous cell carcinoma while TTF-1 expression highlights the benign, entrapped type 2 alveolar pneumocytes. Virtual p40 (d and f) and TTF-1 (i and k) images were synthesised from intensity (d from c and i from h) and FLIM (f from e and k from j) images, showing patterns of expression on an individual cell level almost identical to the ground truth (b vs d and f, and g vs i and k) in both the benign and malignant cell populations l and m show intensity and lifetime contrasts between TTF-1+ and p40+ cells, where histograms were generated from a cluster of those cells. The H&E, p40, and TTF-1 images were acquired from different sequential cuts of the same tissue block.

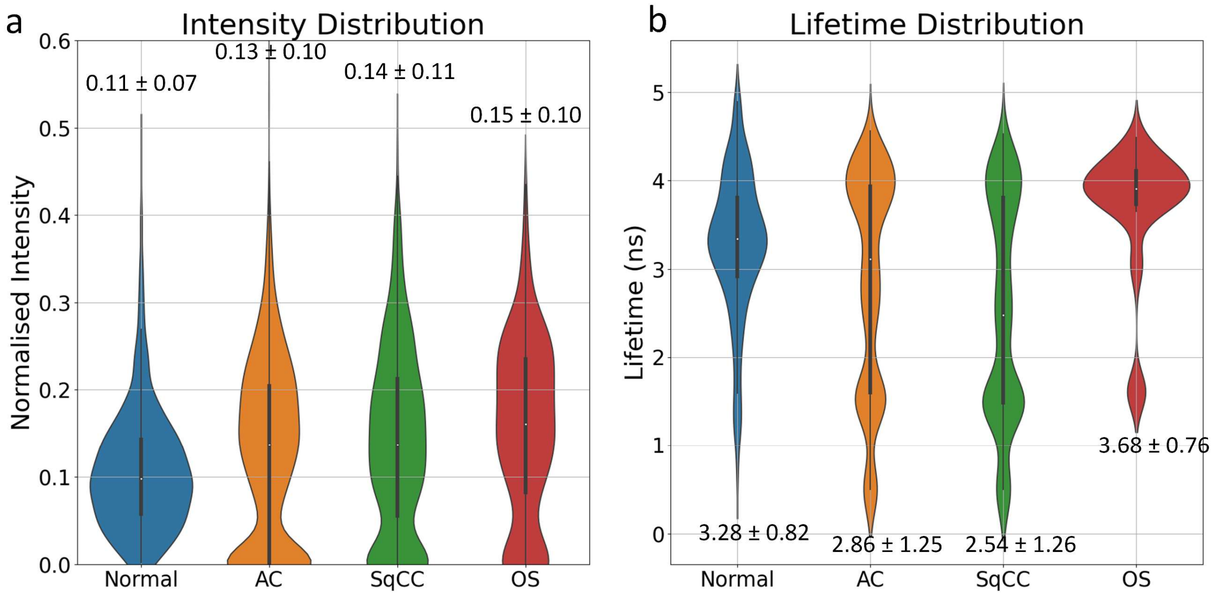

Supplementary Fig. S8. Distributions of means and standard deviations for individual cores of four subtypes, within the test datasets. (a) Normalised intensity value distributions. (b) Lifetime values distributions. The distribution of intensity values is more homogeneous than that of lifetime values.

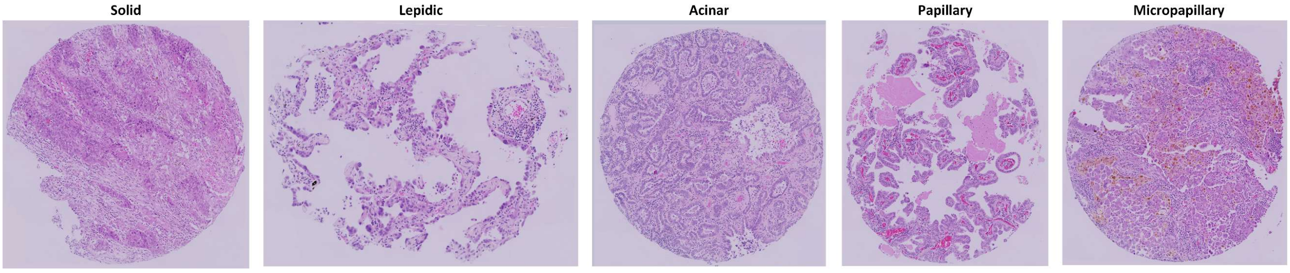

Supplementary Fig. S9. H&E-stained images of AC’s five subtypes, solid, lepidic, acinar, papillary, and micropapillary, involved in the datasets.

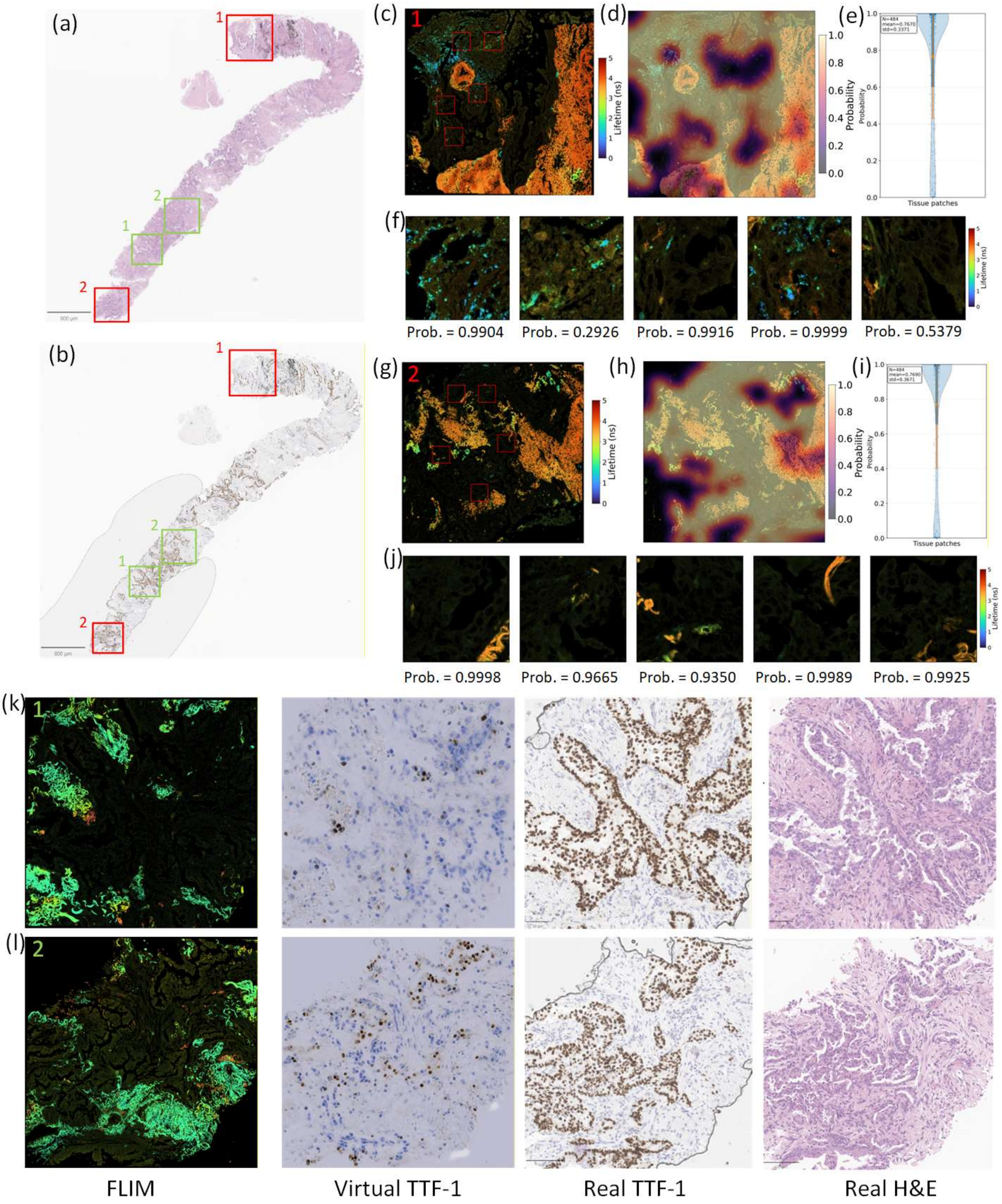

Supplementary Fig. S10. Subtyping and virtual staining results of biopsy 101. H&E overview of the ACC-annotated cancer specimen with regions of interest (ROIs) highlighted (red and green boxes for subtyping and virtual staining) and the corresponding IHC-stained slide are shown in (a, b). FLIM images of selected ROIs from regions 1 and 2 with patch-level sampling locations are shown in (c, g), together with DL predicted subtyping probability heatmaps overlaid on FLIM images in (d, h). The distributions of patch-level prediction probabilities within the ROIs are shown in (e, i), and representative FLIM patches with their corresponding predicted probabilities are shown in (f, j). Qualitative comparisons between virtual TTF-1 staining, real TTF-1 IHC, and real H&E histology for regions 1 and 2 are shown in (k, l).

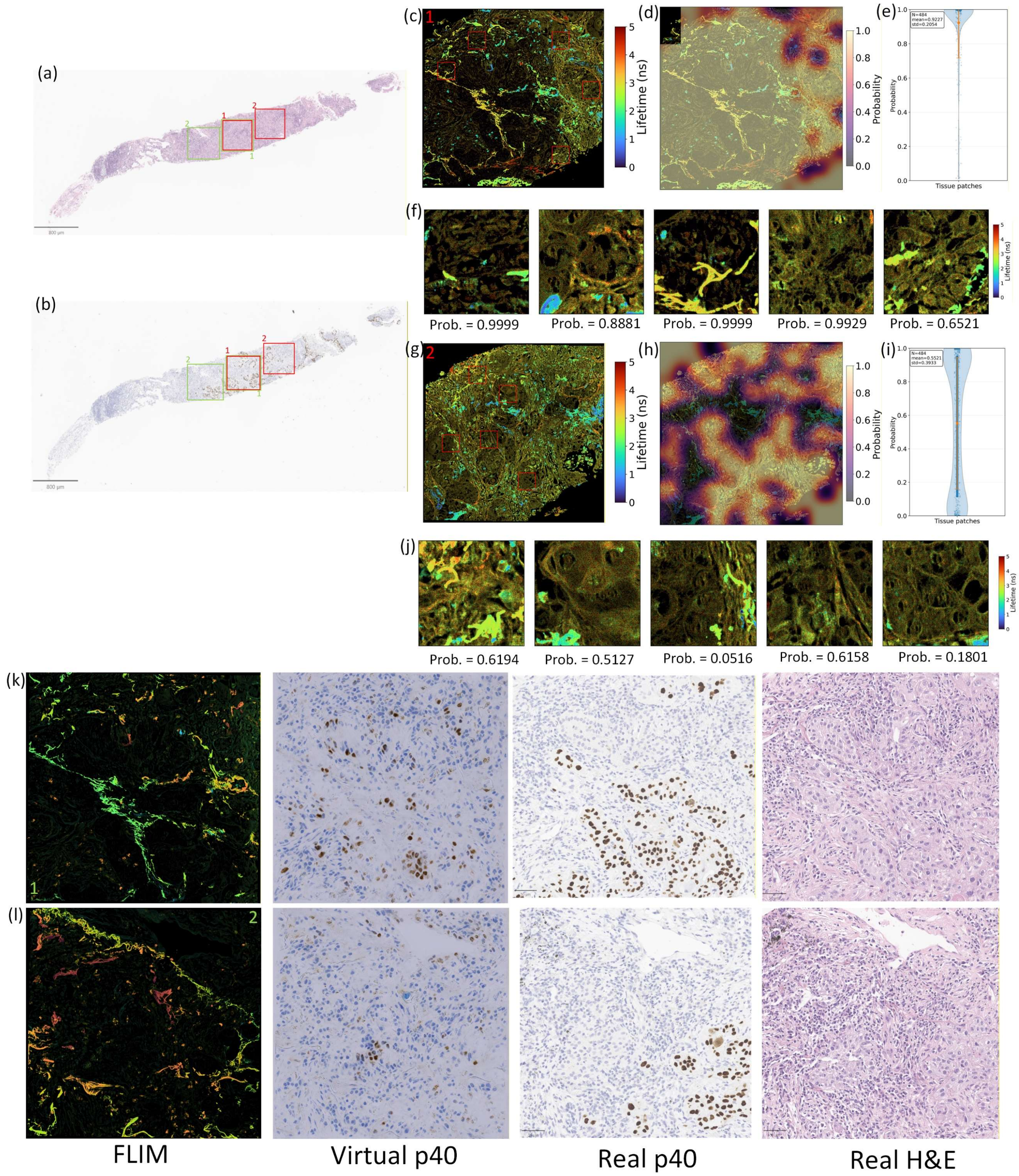

Supplementary Fig. S11. Subtyping and virtual staining results of biopsy 106. H&E overview of the SqCC-annotated cancer specimen with ROIs highlighted (red and green boxes for subtyping and virtual staining) and the corresponding IHC-stained slide are shown in (a, b). FLIM images of selected ROIs from regions 1 and 2 with patch-level sampling locations are shown in (c, g), together with DL–predicted subtyping probability heatmaps overlaid on FLIM images in (d, h). The distributions of patch-level prediction probabilities within the ROIs are shown in (e, i), and representative FLIM patches with their corresponding predicted probabilities are shown in (f, j). Qualitative comparisons between virtual p40 staining, real p40 IHC, and real H&E histology for regions 1 and 2 are shown in (k, l).

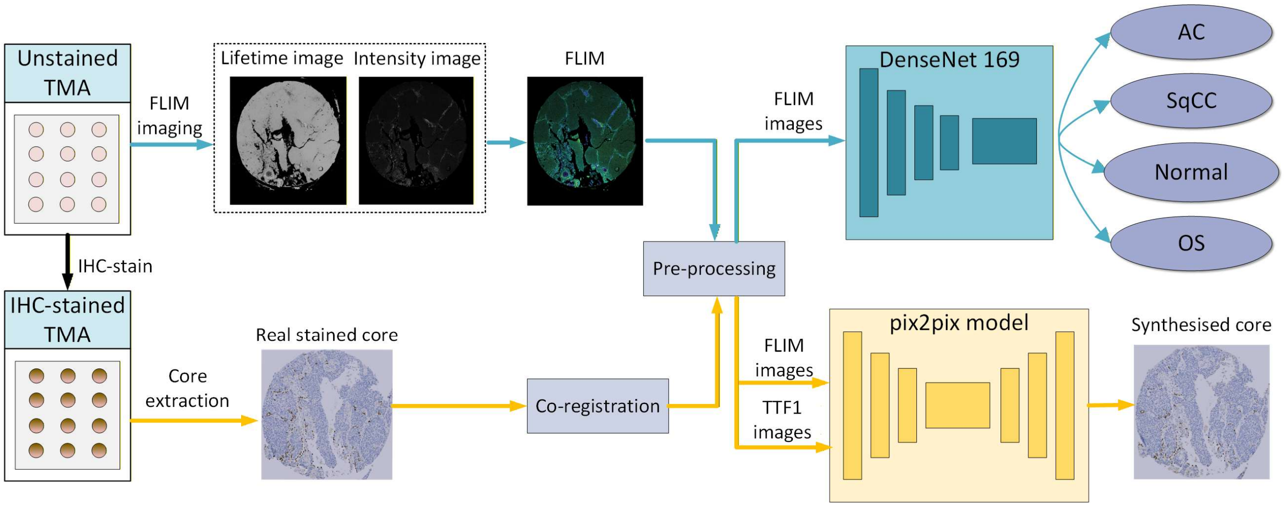

Supplementary Fig. S12. Overview of deep learning architectures for lung cancer subtyping and TTF-1 and p40 virtual staining. Blue and yellow arrows indicate the data processing pipelines for lung cancer subtyping and virtual staining, respectively. The subtyping pipeline consists of FLIM image generation, pre-processing to enhance contrast, and patch generation for downstream deep learning training and inference. The virtual staining pipeline includes co-registration to unify the morphology of FLIM and histology images. The pre-processing module generates paired FLIM and histology patches for training the network.

Table 1. Clinical details on a patient level for the tissue microarrays used in this study.

Demographics	TMA 1 (n=170)	TMA 2 (n=86)	TMA 3 (n=25)
Male, n (%)	72 (42.3%)	40 (46.5%)	12 (48%)
Female, n (%)	98 (57.6%)	46 (53.5%)	13 (52%)
Age, mean (range)	69 (46-92)	67 (44-83)	68 (52-83)
**Smoking Status**			
Current Smoker, n (%)	77 (45.3%)	13 (15.1%)	8 (32%)
Ex-smoker, n (%)	81 (47.6%)	48 (55.8%)	14 (56%)
Non-Smoker, n (%)	10 (5.9%)	25 (29.1%)	3 (12%)
Unknown, n (%)	2 (1.2%)	-	-
**Pathology**			
Adenocarcinoma, n (%)	90 (52.9%)	84 (97.7%)	10 (40%)
Squamous Cell Carcinoma, n (%)	61 (35.9%)	-	10 (40%)
Other- Large Cell Carcinoma, n (%)	7 (4.1%)	-	-
Other- Large Cell Neuroendocrine Carcinoma, n (%)	4 (2.4%)	-	-
Other- Adenosquamous Carcinoma, n (%)	6 (3.5%)	2 (2.3%)	1 (4%)
Other- Pleomorphic Lung Cancer, n (%)	1 (0.6%)	-	-
Other- Adenocarcinoma with Large Cell Neuroendocrine, n (%)	1 (0.6%)	-	1 (4%)
Other- Squamous Cell Carcinoma with Large Cell Neuroendocrine, n (%)	-	-	1 (4%)
Other- Carcinoid, n (%)	-	-	2 (8%)
**Stage (TNM 7)**			
IA, n (%)	54 (31.8%)	-	-
IB, n (%)	54 (31.8%)	-	-
IIA, n (%)	11 (6.5%)	-	-
IIB, n (%)	23 (13.6%)	-	-
IIIA, n (%)	21 (12.4%)	-	-
IIIB, n (%)	7 (4.1%)	-	-
IV, n (%)	0 (0%)	-	-
**Stage (TNM 8)**			
IA1, n (%)	-	2 (2.3%)	0 (0%)
IA2, n (%)	-	7 (8.1%)	5 (20%)
IA3, n (%)	-	5 (5.8%)	2 (8%)
IB, n (%)	-	20 (23.3%)	5 (20%)
IIA, n (%)	-	7 (8.1%)	0 (0%)
IIB, n (%)	-	20 (23.3%)	1 (4%)
IIIA, n (%)	-	19 (22.1%)	6 (24%)
IIIB, n (%)	-	3 (3.4%)	3 (12%)
IVA, n (%)	-	2 (2.3%)	1 (4%)
IVB, n (%)	-	0 (0%)	0 (0%)
N.A.	-	1 (1.2%)	2 (8%)

Table 2. Number of patches and cores of each subtype in training, validation, and test datasets

**No. patches**					
	**AC**	**SqCC**	**OS**	**Normal**	**Total**
**Training**	96,147	82,094	42,820	41,428	262,489
**Validation**	21,328	16,656	7,148	7,904	53,036
**Test**	21,495	13,965	8,181	7,714	51,355
**Total**	138,970	112,715	58,149	57,046	366,880
**No. cores**					
**Training**	188	128	41	85	442
**Validation**	40	27	9	18	94
**Test**	40	27	10	18	95
**Total**	268	182	60	121	631

Table 3. Performance Evaluation of classical deep learning architectures for multiple cancer type classification, using different metrics.

**Multi-Classification**						
	Accuracy Score	Precision Score	Specificity	Recall Score (Sensitivity)	Matthews Correlation Coefficient	AUC Score
**DenseNet-169**	**0.9619**	**0.9621**	**0.9855**	**0.9619**	**0.9458**	**0.9962**
EfficientNet-B0	0.8836	0.8852	0.9564	0.9046	0.8351	0.9925
ResNet-50	0.9467	0.9469	0.9792	0.9467	0.9241	0.9864

Table 4. Ground-truth clinicopathological annotations of lung biopsy specimens, including histological diagnosis and differentiation pattern.

Biopsy index	Diagnosis	Differentiation	Pattern	TTF-1	p40
1	ACC	Moderately	Acinar	Positive	Negative
2	ACC	Poorly	Solid	Positive	Negative
3	ACC	Poorly	Solid, focal acinar	Negative	Negative
4	SqCC	Poorly	Non keratinising	Negative	Positive
5	SqCC	Moderately	Keratinising	Negative	Positive

Table 5. Morphological AC subtypes samples used in this study.

No. Cores										
**AC's subtypes**	**Solid**	**Solid and focal acinar**	**Acinar and Cribriform**	**Lepidic**	**Papillary**	**Acinar and Lepidic**	**Micropapillary**	**Acinar and Micropapillary**	**Acinar**	**Total**
**Amount**	2	2	2	2	2	2	2	2	4	20

Table 6. Extensive Performance Evaluation of DenseNet for binary classification.

	Accuracy	Precision	Specificity	Sensitivity	AUC score	MCC[1]
**Cancer & non-Cancer**	0.9984	0.9979	0.9975	0.9991	0.9967	1.0000
**AC & SqCC + OS**	0.9310	0.9346	0.9372	0.9246	0.8621	0.9807
**SqCC & OS**	0.9804	0.9771	0.9603	0.9923	0.9580	0.9982
**AC & SqCC**	0.8935	0.9070	0.8550	0.9184	0.7762	0.9607

1. Matthews Correlation Coefficient
